# GENDER AND BELIEFS ABOUT SUCCESSFUL AGING IN EASTERN NEPAL

**DOI:** 10.1093/geroni/igz038.3131

**Published:** 2019-11-08

**Authors:** Xiao Qiu, Na Sun, Sara J McLaughlin, Janardan Subedi, Suman S Thapa, Mohan K Shrestha, Matthew Johnson, Sarah Williams-Blangero

**Affiliations:** 1 Miami University, Oxford, Ohio, United States; 2 Tilganga Institute of Ophthalmology, Kathmandu, Kathmandu, Nepal; 3 Tilganga Institute of Ophthalmology, Kathmandu, Nepal; 4 The University of Texas Rio Grande Valley, Brownsville, Texas, United States

## Abstract

Gender shapes opportunities and experiences over the life course, which may influence beliefs about what it means to age successfully. In Nepal, a developing nation in South Asia, women and girls have historically had fewer social and economic opportunities than their male counterparts. To understand how gender may shape beliefs about successful aging, adult members of the Jiri population in eastern Nepal were asked to rate the importance of health-related (e.g., longevity), psychological (e.g., satisfaction with life), and social (e.g., support of family and friends) elements of successful aging (n = 1479; 52.9% female; 49.0% age 18-39, 33.1% age 40 to 59, 17.8% age 60 and over). Each of the 13 elements was rated as very important by over two thirds of the sample. Few gender differences in beliefs were observed; however, results of logistic regression analysis indicate that the odds of Jiri women endorsing longevity (OR = 0.75, p = 0.02) and life satisfaction (OR = 0.65, p = 0.02) as very important to successful aging were significantly lower than for men, controlling for age, education, and presence of illness. While more similarities than differences in beliefs about successful aging were observed by gender, the extent to which socially-defined roles and expectations may be responsible for observed differences should be explored in future research.

